# Integrated circulating tumour DNA and cytokine analysis for therapy monitoring of ALK-rearranged lung adenocarcinoma

**DOI:** 10.1038/s41416-023-02284-0

**Published:** 2023-04-29

**Authors:** Arlou Kristina Angeles, Florian Janke, Ann-Kathrin Daum, Martin Reck, Marc A. Schneider, Michael Thomas, Petros Christopoulos, Holger Sültmann

**Affiliations:** 1 Division of Cancer Genome Research, German Cancer Research Center (DKFZ), German Cancer Consortium (DKTK), and National Center for Tumor Diseases (NCT), Heidelberg, Germany; 2 German Center for Lung Research (DZL), TLRC Heidelberg, Heidelberg, Germany; 3 Lung Clinic Grosshansdorf, Airway Research Center North, German Center for Lung Research, Grosshansdorf, Germany; 4 Translational Research Unit, Thoraxklinik at University Hospital Heidelberg, Heidelberg, Germany; 5 Department of Oncology, Thoraxklinik at University Hospital Heidelberg, Heidelberg, Germany

**Keywords:** Non-small-cell lung cancer, Tumour biomarkers

## Abstract

**Background:**

Detection of circulating tumour DNA (ctDNA) in biological fluids is a minimally invasive alternative to tissue biopsy for therapy monitoring. Cytokines are released in the tumour microenvironment to influence inflammation and tumorigenic mechanisms. Here, we investigated the potential biomarker utility of circulating cytokines vis-à-vis ctDNA in ALK-rearranged+ lung adenocarcinoma (ALK + NSCLC) and explored the optimal combination of molecular parameters that could indicate disease progression.

**Methods:**

Longitudinal serum samples (*n* = 296) were collected from ALK + NSCLC patients (*n* = 38) under tyrosine kinase inhibitor (TKI) therapy and assayed to quantify eight cytokines: IFN-γ, IL-1β, IL-6, IL-8, IL-10, IL-12p70, MCP1 and TNF-α. Generalised linear mixed-effect modelling was performed to test the performance of different combinations of cytokines and previously determined ctDNA parameters in identifying progressive disease.

**Results:**

Serum IL-6, IL-8 and IL-10 were elevated at progressive disease, with IL-8 having the most significant impact as a biomarker. Integrating changes in IL-8 with ctDNA parameters maximised the performance of the classifiers in identifying disease progression, but this did not significantly outperform the model based on ctDNA alone.

**Conclusions:**

Serum cytokine levels are potential disease progression markers in ALK + NSCLC. Further validation in a larger and prospective cohort is necessary to determine whether the addition of cytokine evaluation could improve current tumour monitoring modalities in the clinical setting.

## Introduction

Tumorigenesis and the development of metastatic disease are supported not only by the acquisition of clonal genetic and epigenetic alterations. The outgrowth of tumour cells is also influenced by the tumour microenvironment (TME), which hosts other non-transformed stromal cells such as fibroblasts, endothelial cells, mesenchymal stem cells, and immune cells [1–3]. The TME facilitates the secretion of a variety of cytokines that may ameliorate tumour growth or promote chronic inflammation, leading to poor disease outcomes [4]. As cytokines are detectable in circulation, they are potentially useful for minimally invasive liquid biopsies, in addition to other cancer-specific biomarkers such as circulating tumour DNA (ctDNA) [5]. Previous studies have shown significant associations of serum cytokine levels with the risk for tumorigenesis in patients of different cancers such as lung [6, 7], ovarian [8], breast [9, 10] and colorectal [11, 12] carcinoma compared to healthy controls.

Approximately 3–7% [13] of non-small cell lung cancers harbour a structural rearrangement in the *ALK* gene (ALK + NSCLC) resulting in the constitutive activation of the translated protein. The development of ALK inhibitors for targeted therapy has improved the prognosis of ALK + NSCLC patients. However, treatment resistance eventually develops, commonly through the emergence of secondary *ALK* resistance mutations and other acquired alterations [14]. Close monitoring of therapy success or failure is thus critical for patient management. While tumour rebiopsies could detect the emergence of clinically relevant tumour clones, these procedures are associated with procedural risk that precludes their use in many cases. On the other hand, liquid biopsy is a minimally invasive alternative for disease monitoring, which can detect ctDNA from plasma. Liquid biopsy technologies can capture information coming from the primary tumour as well as metastatic sites, which addresses the limitation posed by tumour heterogeneity in conventional tissue biopsies [15]. We have previously [16, 17] applied next-generation sequencing (NGS) assays on plasma DNA in a retrospective ALK + NSCLC cohort and quantified ctDNA levels by variant allele frequencies (VAF_mean_) of single nucleotide variants and t-MAD scores [18], which inform gross copy number changes across the genome. We found that ctDNA abundance is associated with tumour progression, and has the capacity to identify early molecular progression.

Recently, the prognostic significance of systemic inflammation-based prognostic scores in ALK + NSCLC patients under first-generation ALK inhibitor therapy has been reported [19]. In this study, we aimed to explore the relevance of serum cytokine levels for longitudinal patient monitoring in ALK + NSCLC. A selection of cytokines was quantified using a multiplex immunoassay in a subset of the ALK + NSCLC cohort from which longitudinal ctDNA data were available [16, 17]. Furthermore, we integrated previously published ctDNA data and cytokine datasets generated in the current study to determine the optimal combination of multi-analyte classifiers that can discriminate between stable and progressive disease.

## Methods

### Patients and sample collection

**Fig. 1:**
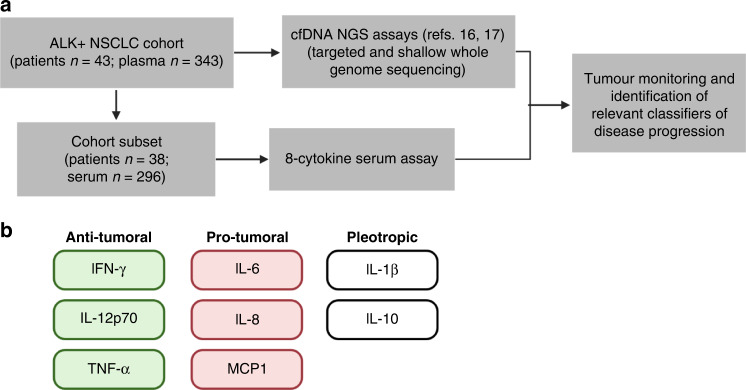
Serum cytokine analysis was performed in longitudinal samples in ALK + NSCLC patients. **a** Patient cohort description and summary of workflow. **b** The cytokines analysed in this study, grouped according to reported function in tumour biology.

The ethics committees of Heidelberg University (S-270/2001, S-296/2016) and Lübeck University (AZ 12-238) approved the study. Written informed consent was obtained from all study participants. Patient characteristics are detailed in Table 1 and Supplemental Fig. 1. Peripheral blood was collected through venipuncture at each outpatient visit at Thoraxklinik Heidelberg and Lungenclinic Grosshansdorf, Germany. Blood was collected in lithium-heparin tubes, and within 1 h of the blood draw, serum was separated by centrifugation at 2000×*g* for 10 min. Serum samples were stored at −80 °C in the Lung Biobank Heidelberg/BMBH until further processing. Thirty-eight patients from the original cohort were included for cytokine analysis, corresponding to 296 longitudinal serum samples (Fig. 1a). Clinical data, routine radiographic assessments (every 8–12 weeks) using chest/abdominal computerised tomography and brain MRI, as well as routine laboratory results, specifically complete blood counts, serum lactate dehydrogenase (LDH) and serum C-reactive protein (CRP) levels, were collected through a review of patient records with a cut-off date of September 15, 2020. Diagnosis of ALK + NSCLC with analysis of the *ALK* fusion variant and *TP53* status were based on DNA and RNA next-generation sequencing lung cancer-specific gene panels on the IonTorrent platform, as published [20]. Briefly, tumour tissue was micro-dissected to achieve histological tumour content of at least 15%, and nucleic acid extraction was performed using the Maxwell 16 LEV DNA kit or RNA FFPE Purification kit (Promega, Madison, USA) following the manufacturer’s protocol. Sequencing libraries were generated using the multiplex PCR-based Ion Torrent AmpliSeq^TM^ technology in conjunction with the RNA Lung Cancer Fusion Panel and the Lung Cancer Panel (Thermo Fisher Scientific, Waltham, USA). The Ion Torrent Suite Software (version 5.0 up to 5.6) was used to process raw sequencing data. The built-in Variant Caller plugin (version 5.0 up to 5.6) was used for DNA mutation analysis. The fusion workflow integrated into the Ion Reporter Software (version 5.0) was used for the detection of fusion transcripts.

**Table 1:** Patient characteristics.

ALK + NSCLC patients analysed in this study (*n* = 38)		
Age, median (range)		59 (39–80)
Sex, % male		53%
Smoking status (% never smokers)		79%
ECOG PS (%) at baseline	0	26
	1	14
	2	1
	No data	2
Histology	Adenocarcinoma^a^	37/38
*ALK* fusion variant^b^	*EML4-ALK* V3	11
	*EML4-ALK* V1/V2	22
	Other	4
	No data	1
*TP53* status at baseline, mutated^c^		9/36
ALK TKI, patient number	Crizotinib	23
	Ceritinib/alectinib/brigatinib	32
	Lorlatinib	4
Chemotherapy		7
Follow-up in months (median [Q1-Q3])		38 (26–52)
Number of samples analysed per patient (median [range])		8 (3–24)
Number of TKI lines covered with LiBx per patient, mean		2.1
Number of samples at disease progression with therapy change per patient, mean		0.95

*EML4-ALK* echinoderm microtubule-associated protein-like 4 (*EML4*) and anaplastic lymphoma kinase (*ALK*) fusion, *PS* performance status, *TKI* tyrosine kinase inhibitor, *Q1* quartile 1, *Q3* quartile 3, *LiBx* liquid biopsy.

^a^One patient had an ALK^+^ large-cell neuroendocrine lung carcinoma responsive to ALK inhibitors.

^b^Data available for 37/38 cases; one case with E18A20, one with E9A20, one with K9A20 (*KLC1*), and one with K24A20 (*KIF5B*).

^c^Data available for 36 cases by NGS of tissue biopsies at diagnosis of Stage IV disease.

### NGS assays for plasma DNA

ctDNA data used in this study were generated using capture-based targeted sequencing and shallow whole genome sequencing (sWGS), as previously reported [16, 17]. Briefly, sequencing libraries were prepared using the AVENIO ctDNA Library Preparation Kit with either the Targeted or Surveillance Panel (Roche Diagnostics). Library pools were sequenced on the Illumina NextSeq 550 platform with the High-Output Kit V2 (2 × 150 bp). Downstream analysis was performed using the AVENIO ctDNA analysis software (Roche Diagnostics, version 2.0.0), applying a variant allele frequency threshold of 0.01%. In parallel, libraries for sWGS were prepared using the KAPA HyperPrep Kit with KAPA Dual-Indexed Adaptors, and sequenced on the Illumina HiSeq 4000 platform (2 × 100 bp). Genome-wide copy number profiles were estimated using ichorCNA [21]. Trimmed Median Absolute Deviation from copy number neutrality (t-MAD) scores were calculated as previously described [17, 18].

### Cytokine selection and measurement

Serum cytokines were quantified using the FirePlex®-96 Inflammation Human Immunoassay Panel (Abcam, Cambridge, UK), which multiplexes the detection of eight cytokine targets: IFN-γ, IL-1β, IL-6, IL-8, IL-10, IL-12p70, MCP1 and TNF-α (Fig. 1b). Published evidence of the serum biomarker potential of all 8 cytokines in NSCLC is available [22]. IFN-γ initiates antitumor processes by activating JAK-STAT signalling, and the transcription of IFN-γ-inducible genes resulting in cell-cycle arrest and apoptosis [23]. IL-1β is a pleiotropic cytokine that correlates with tumour progression in NSCLC, and is associated with poor survival [24, 25]. Serum IL-6 and IL-8 are associated with a higher risk of lung cancer [6] and lung cancer mortality [26]. IL-10 is another pleiotropic cytokine that has been shown to be increased in the serum and bronchoalveolar lavage fluid of NSCLC patients, with higher abundance in advanced disease [27]. The antitumor activities of IL-12p70 include induction of IFN-γ production [28], stimulation of growth and cytotoxicity of activated NK cells, CD8 + and CD4 + T cells against tumour cells [29]. Nonetheless, circulating IL-12p70, together with IL-6 and IL-8, was reported to be elevated in NSCLC patients compared to control subjects [30]. MCP1 is a chemokine with potent monocyte chemotactic activity. Increased MCP1 in NSCLC leads to higher tumour infiltrating macrophages [31]. TNF-α is an established modulator of inflammation and cell death processes [32]. It was first reported as a serum factor that promoted tumour necrosis [33]. Interestingly, more recent studies have associated TNF-α in promoting resistance to targeted treatment and immunotherapy [34, 35]. Cytokine analysis was performed following the manufacturer’s protocol. Serum samples were diluted 1:2 with the human assay diluent prior to analysis and a fivefold dilution of protein standards was adapted in this study. Each serum sample was assayed in duplicate. Fluorescent measurements were acquired using the InCyte 3.3 software and the Guava easyCyte HT flow cytometer (Merck Millipore, Darmstadt, Germany). Serum concentration analysis was performed using the Fireplex Analysis Workbench version 2.2.274-ext. Quantification of serum levels for IL-6, IL-8 and IL-10 in healthy controls and non-ALK NSCLC patients was performed within another study as published before [22].

### Statistical analysis

Serum cytokine differences between stable (SD) and disease progression accompanied by therapy change (PDTC) were tested using the Mann–Whitney *U* test. Longitudinal samples, even those sampled from the same patient, were analysed independently as done previously [16]. For three or more groups, statistical significance was tested using nonparametric Kruskal–Wallis, followed by multiple comparisons. Survival data were analysed using the log-rank test, using the median of cytokine value as threshold for high and low groups. These tests and all Spearman correlation analyses were performed using GraphPad Prism (ver. 9.4.0). The percent change in cytokine values was calculated as the difference between two successive sampling points, divided by the cytokine measurement of the earlier time point (i.e., t_1_), as shown in the following equation:

$${{{{{{{\mathrm{\% }}}}}}}}\Delta cytokine = \left( {\frac{{cytokine_{t_2} - cytokine_{t_1}}}{{cytokine_{t_1}}}} \right) \times 100{{{{{{{\mathrm{\% }}}}}}}}$$

Generalised linear mixed-effect modelling (GLMM) was performed to test the different combinations of cytokines and ctDNA parameters in distinguishing SD and PDTC samples. In addition to accommodating binary outcomes, this approach also allows the inclusion of random patient effects inherent in longitudinal sampling [36]. The R package lme4 (ver. 1.1-23) was used for model fitting. The R package pROC (ver. 1.16.2) was used to generate receiver operating characteristic (ROC) curves, estimate areas under the curve (AUC), and to compare the AUCs of two ROC curves. The 95% confidence intervals of the AUC were computed with 2000 stratified bootstrap replicates. ROC curve comparison was performed using the DeLong method [37]. R-based analyses were performed on RStudio (ver. 1.2.1335) with R (ver. 4.0.2).

## Results

### Serum levels of IL-6, IL-8 and IL-10 are elevated at progressive disease

A subset from a previously reported metastatic ALK + patient cohort [16] was included in the current study. In total, 296 longitudinal serum samples were retrospectively collected from 38 patients for cytokine analysis. All cytokines were detectable within the dynamic range of the multiplex assay. The physiological serum abundance of each cytokine was distinct with the following median values in pg/mL: IL-12p70, 0.5591 (range: 0.05973–12.43); IL-1β, 0.9400 (range: 0.1032–15.39); IFN-γ, 2.960 (range: 0.1041–22.42); IL-6, 4.980 (range: 0.5709–265.5); IL-10, 7.958 (range: 2.656–38.57); TNF-α, 8.496 (range: 4.193–22.60); IL-8, 14.21 (range: 1.727–251.2); MCP1, 299.3 (range: 76.81–1800) (Supplementary Fig. 2a). There were strong positive correlations among IFN-γ, IL-1β, and IL-12p70 (Spearman ρ_IFN-γ vs. IL-1β_ = 0.63, ρ_IL-1β vs. IL-12p70_ = 0.57, ρ_IFN-γ vs. IL-12p70_ = 0.56) (Supplementary Fig. 2b). Moderate correlations (ρ < 0.5) were present between IL-6 and IL-10 (ρ = 0.46), IL-8 and MCP1 (ρ = 0.45), IL-10 and TNF-α (ρ = 0.43) as well as IL-6 and IL-8 (ρ = 0.36).

**Fig. 2:**
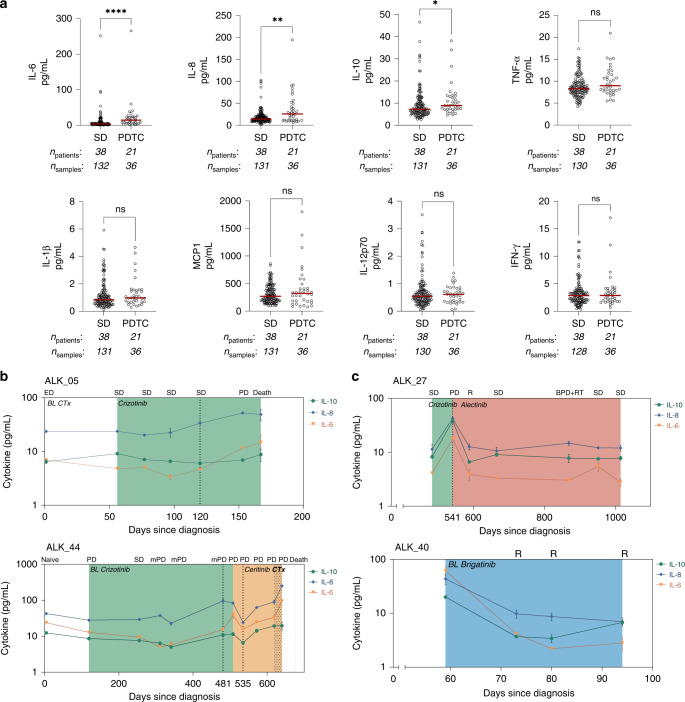
IL-6, IL-8 and IL-10 serum levels are elevated at disease progression. **a** Comparisons of serum cytokine levels in stable and progressive disease in the ALK + NSCLC cohort. Each dot represents the median of duplicate measurements per sample. The median of each group is shown by the red line. Patient counts and corresponding serum samples used in each group are indicated below the *x* axis. Statistical significance was tested using Mann–Whitney *U* test, ns not significant; *P < 0.05; **P < 0.01; ***P < 0.001. Longitudinal serum cytokine kinetics of representative patients illustrating that (**b**) increase in cytokine levels reflect tumour progression while (**c**) lowering or consistent values were obtained during stable disease and therapy response. Time points mentioned in the main text are indicated by broken lines. CTx chemotherapy, SD stable disease, PDTC progressive disease with therapy change, mPD metastatic progressive disease, BPD brain progressive disease, RT radiotherapy, R response.

Among the eight cytokines measured, IL-6, IL-8 and IL-10 showed significantly increased serum abundance in samples collected at disease progression accompanied by a therapy change (PDTC), compared to serum sampled at stable disease (Fig. 2a). We investigated if the baseline serum levels of these cytokines could inform therapy duration in terms of progression-free survival (PFS). While baseline IL-8 had a tendency to stratify the length of PFS, this did not reach the threshold of significance (Supplementary Fig. 3). Baseline IL-6 and IL-10 were also not predictive of PFS. We then inspected the kinetics of these three cytokines alongside the clinical status determined by radiographic imaging to evaluate their utility for tumour monitoring. We found cases where IL-6, IL-8 and IL-10 showed increasing trends upon disease progression (Fig. 2b). For patient ALK_05 under crizotinib treatment, serum IL-6 and IL-8 increased even before progressive disease was evaluated (120 days since diagnosis), and continued to increase until disease progression (153 days since diagnosis). Under crizotinib therapy, patient ALK_44 similarly showed elevated cytokine levels at disease progression (481 days since diagnosis). After switching to ceritinib, aside from an initial drop at day 535, the cytokines continually increased in the serum alongside tumour progression until the last available serum sample prior to patient death. Notably, these trends were in agreement with the ctDNA parameters VAF_mean_ and t-MAD scores previously analysed (Supplementary Fig. 4).

Simultaneously, we also observed cases where cytokine levels did not vary across time points with stable disease (Fig. 2c). Patient ALK_27 under alectinib therapy was a representative case. After a progressive disease point (541 days since diagnosis) where IL-6, IL-8 and IL-10 abundance sharply increased, the cytokine levels fell considerably at treatment response and remained at a constant level throughout the stable disease points. Notably, the cytokine abundance did not change at an intermittent point of brain progression with radiotherapy. Patient ALK_40 who underwent brigatinib treatment exemplified reduction of serum cytokine levels upon treatment response. The last sampling point revealed slight increases in the levels of IL-6 and IL-10. However, the relevance of these changes could not be assessed due to the lack of further samples. With the exception of the VAF_mean_ trend of patient ALK_40, ctDNA parameters had the tendency of abundance similar to serum cytokines (Supplementary Fig. 4). The patients described illustrate the potential of serum IL-6, IL-8 and IL-10 for tumour monitoring in ALK + NSCLC.

We previously reported that serum IL-6, IL-8, and IL-10 levels are elevated in metastatic non-ALK NSCLC compared to healthy controls [22]. By integrating these data with the results of the current work, we also observed that ALK + NSCLC patients that were treatment naive or at therapy baseline have significantly elevated serum IL-6, IL-8 and IL-10 levels compared to healthy controls (Supplementary Fig. 5). Serum cytokine levels in ALK+ patients were also significantly higher compared to non-ALK patients. However, this might be due to the representation of both naive and pre-treated samples in the ALK+ cohort, as well as technical differences between studies. Nonetheless, these observations suggest that IL-6, IL-8 and IL-10 could be useful markers for NSCLC in general.

**Fig. 3:**
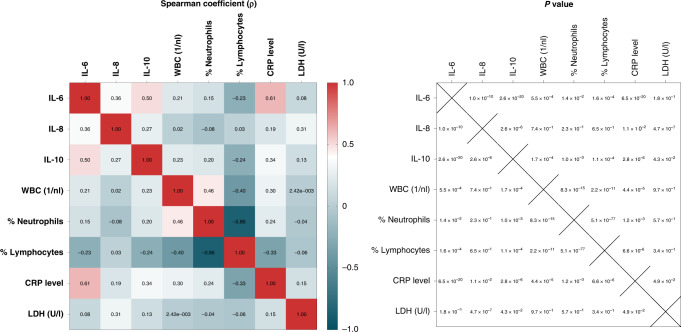
IL-6, IL-8 and IL-10 are weakly correlated with clinical markers of systemic inflammation. Spearman correlation coefficient matrix (left) and corresponding p-values (right) showing associations between cytokines and other clinical markers of systemic inflammation.

As cytokine release could be influenced by tumour unspecific factors such as infections and steroid administration, we compared clinical markers of systemic inflammation—particularly white blood cell (WBC) count, blood neutrophil percent (%neutrophil), blood lymphocyte percent (%lymphocyte), CRP and LDH—with serum IL-6, IL-8 and IL-10 levels to assess possible associations (Fig. 3). The only strong correlation between any of the cytokine and inflammation markers was that of IL-6 and CRP (ρ = 0.61), most likely due to the known transcriptional induction of the protein by IL-6 [38]. There were only weak associations between IL-10 and CRP (ρ = 0.34), and between IL-8 and LDH (ρ = 0.31). These suggest that IL-6, IL-8 and IL-10 are not simply molecular surrogates of—and could contribute independent information beyond—the systemic inflammation status of the patient. Besides, serum IL-6 and IL-8 levels were higher in patients with *TP53* mutation compared to *TP53* wild-type tumours, while serum IL-6 and IL-10 levels were higher in patients with *EML4-ALK* variant 3 (V3) compared to V1/V2-driven tumours (Supplementary Fig. 6), indicating an association between serum cytokines levels and molecular risk factors of ALK + tumours.

### IL-6, IL-8 and IL-10 are tumour progression markers independent of ctDNA abundance

**Fig. 4:**
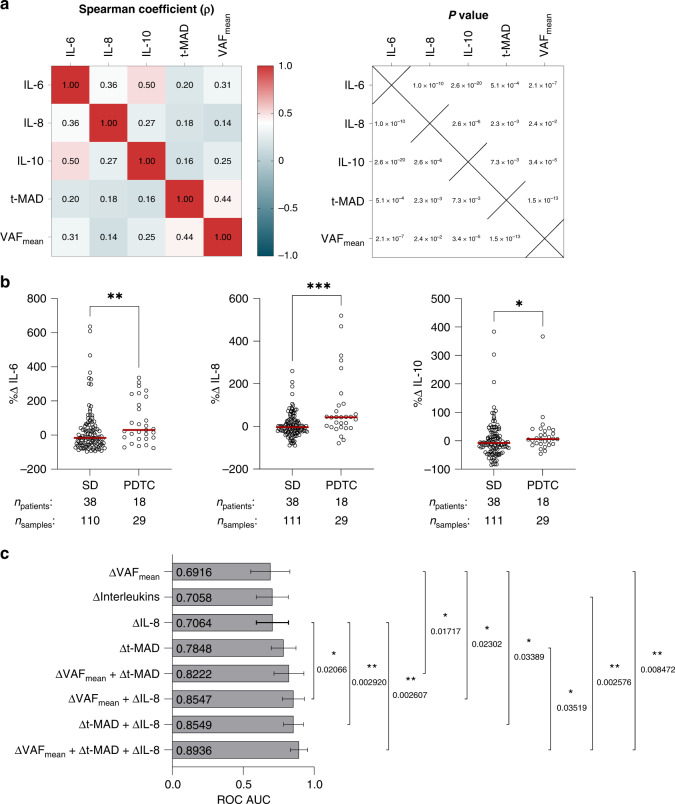
Combinatorial analysis of serum cytokines and ctDNA parameters for identifying disease progression in ALK + NSCLC. **a** Spearman correlation coefficient matrix (left) and corresponding *P* values (right) showing associations between cytokines and ctDNA parameters, t-MAD score and VAF_mean_. **b** Comparisons of changes in serum cytokine levels in stable and progressive disease. The median of each group is shown by the red line. Patient counts and corresponding serum samples used in each group are indicated below the *x* axis. Statistical significance was tested using Mann–Whitney *U* test, ns not significant; *P < 0.05; **P < 0.01; ***P < 0.001. **c** Area under the curve (AUC) values of receiver operating characteristic (ROC) curves distinguishing stable and progressive disease in ALK + NSCLC using independent and combinatorial liquid biopsy parameters. Pairwise AUC significance test was performed using the DeLong method. ΔInterleukins: ΔIL-6 + ΔIL-8 + ΔIL-10. Error bars indicate the 95% confidence intervals. Only significant comparisons are shown. *P < 0.05; **P < 0.01; ***P < 0.001.

We have previously established the utility of ctDNA detection for tumour monitoring in ALK + NSCLC using targeted panel and shallow whole genome sequencing-based assays [16, 17]. CtDNA abundance was quantified based on these analyses using the VAF_mean_, which is the average of all detected variant allele frequencies, and the t-MAD score, which estimates the gross copy number changes genome-wide. To determine the redundancy of serum cytokine abundance for tumour monitoring, we checked the correlations of IL-6, IL-8 and IL-10 with these two ctDNA parameters. Strong correlations were absent between the cytokines and ctDNA markers (Fig. 4a), suggesting that the serum cytokine analysis can discriminate stable and progressive disease independent of ctDNA abundance.

### Change in serum IL-8 is informative of tumour progression and complements ctDNA analysis for distinguishing disease status

To minimise the effects of patient heterogeneity and patient-dependent therapeutic schemes that could influence the physiological baseline levels of serum cytokines [39], we proceeded with our analysis using the change of cytokine abundance between two consecutive time points instead of using absolute cytokine values. We reasoned that a considerable change in the analyte level is more capable of capturing the dynamic clinical status of a patient, compared to measurements taken at each time point which could be inherently high or low depending on the individual patient. This was an approach similarly taken in evaluating the utility of ctDNA monitoring in the same cohort [16], and in other studies [40, 41] that applied ctDNA for disease monitoring. We took the percent change of all cytokines at consecutive time points and found that IL-6, IL-8 and IL-10 were still the most relevant indicators of disease progression (Fig. 4b). Changes in the remaining cytokines were not significantly different between time points leading to stable or progressive disease (Supplementary Fig. 7).

Given the potential of ΔIL-6, ΔIL-8 and ΔIL-10 for identifying the progressive disease, and their poor correlation with ctDNA parameters, we determined the optimum combination of these liquid biopsy parameters by fitting generalised linear mixed-effects models, accounting for patient effects since longitudinal samples were obtained from each patient (Fig. 4c). ΔVAF_mean_ alone could distinguish disease status with a ROC AUC of 0.6916 (range: 0.5536–0.8296). The ability of serum cytokines (ΔInterleukins) to indicate tumour status was illustrated by its almost equal AUC (0.7058, range: 0.5930–0.8186) to ΔVAF_mean_. Δt-MAD score had a ROC AUC of 0.7848 (range: 0.6981–0.8716). Next, the combination of all three cytokines yielded a ROC AUC of 0.7058 (range: 0.5930–0.8186), with IL-8 the cytokine with the most significant effect on the response (stable vs. progressive disease, *P*_ΔIL-6_ = 0.97996, *P*_ΔIL-8_ = 0.00304, *P*_ΔIL-10_ = 0.98048). Moreover, ΔIL-8 on its own yielded a slightly higher ROC AUC (0.7058, range: 0.5930–0.8186) than all three interleukins combined. Given these results, we then tested ΔIL-8 in combination with ΔVAF_mean_ and/or Δt-MAD to determine whether ΔIL-8 in the serum could provide additional information to ctDNA parameters for tumour monitoring. ΔIL-8 and ΔVAF_mean_ together yielded a ROC AUC of 0.8547 (range: 0.7774–0.9320), which is a significant improvement over the AUC of ΔVAF_mean_ alone (*P* = 0.02302). On the other hand, while the combination of ΔIL-8 and Δt-MAD slightly increased the AUC to 0.8549 (range: 0.7856–0.9241), this did not result in a significant improvement of marker performance (*P* = 0.1277). The addition of ΔVAF_mean_ to the bivariate model of ΔIL-8 and Δt-MAD however, significantly outperformed (*P* = 0.03519) the Δt-MAD marker alone, resulting in an AUC of 0.8936 (range: 0.8325–0.9546). Notably, the combination of both ctDNA markers (AUC = 0.8222, range: 0.7168–0.9277) did not significantly improve (*P* = 0.2347) with the addition of ΔIL-8 despite maximising the AUC. Also noteworthy was ΔIL-8 alone is sufficient as a cytokine classifier and additional ΔIL-6 and ΔIL-10 data did not significantly affect the resulting AUCs (Supplementary Fig. 8). Altogether, these results suggest that while comprehensive ctDNA analysis (i.e., using ΔVAF_mean_ and Δt-MAD) remains superior for tumour monitoring in ALK + NSCLC, the information provided by serum cytokines, particularly of ΔIL-8, is independent and complementary to ctDNA mutational analysis data.

## Discussion

The development of ALK inhibitors—from the first-generation tyrosine kinase inhibitor (TKI) crizotinib and next-generation inhibitors alectinib, ceritinib, brigatinib and lorlatinib—improved the prognosis of ALK + NSCLC patients through prolonged progression-free survival and better response rates, with enhanced drug penetration into the central nervous system [42]. Nonetheless, patients develop therapy refraction and subsequently suffer poor clinical outcomes if not assessed and managed in a timely manner. Disease monitoring in ALK + patients using ctDNA is a minimally invasive alternative to tissue rebiopsies and can potentially be used for real-time monitoring of disease and therapy response [43]. We [16, 17, 44] and others [45, 46] have shown that ctDNA analysis in ALK + NSCLC can detect tumour-specific genetic alterations and copy number changes which correlate with tumour burden and clinical outcome. Although it is feasible for ctDNA analysis to survey the heterogeneity of the tumour genome at multiple time points, this requires significant technical expertise and resources [15].

Numerous studies [6, 26, 47, 48] have shown that inflammatory factors contribute to the aetiology of several human cancers. In lung malignancies, circulating immune and inflammation markers have been utilised for prospective risk stratification studies [6, 7, 26]. Other reports revealed the significance of inflammation markers in predicting NSCLC survival, particularly in patients with *EGFR*-mutant tumours treated with tyrosine kinase inhibitors [49, 50]. The pan-immune-inflammation value, which is based on peripheral blood cell counts, was recently used in a retrospective study for predicting the progression-free- and overall survival of ALK + NSCLC patients undergoing first-line ALK TKI therapy [51]. On the other hand, the tumour monitoring value of circulating cytokine markers in ALK + NSCLC has yet to be explored and remains unclear. Here, we investigated the utility of circulating cytokines, which are easily accessible serum analytes, for tumour monitoring in ALK + NSCLC, and evaluated the orthogonal merit of cytokine analysis to ctDNA in a multi-analyte liquid biopsy approach.

Among the cytokines tested, the most significant indicators of tumour progression in ALK + NSCLC were IL-6, IL-8 and IL-10. These cytokines are also elevated in metastatic non-ALK NSCLC patients at therapy baseline compared to healthy controls [22]. We provided evidence that these cytokines were more abundant in ALK + NSCLC patients compared to healthy controls, suggesting that IL-6, IL-8 and IL-10 could be relevant serum markers for NSCLC, as reported in other independent studies [6, 26, 27]. However, we could not perform a direct comparison between non-ALK and ALK + NSCLC cases due to differences in cohort composition (e.g., treatment status). Cytokine measurement methodologies, including cytokine panel platforms used, were also study-dependent. Future prospective studies are necessary to compare the relative cytokine levels between NSCLC subtypes.

Serum IL-6 and IL-8 were previously reported to increase the risk of lung cancer [6], and were significantly associated with tumour recurrence in Stage I lung cancer patients treated with surgery [26]. IL-6 is a pro-inflammatory cytokine, promotes tumorigenic processes and activates PI3K–AKT, mitogen-activated protein kinase (MAPK)/extracellular signal-regulated kinase (ERK), NF-κB and STAT3 signalling [4]. IL-8 mediates an immunosuppressive TME, facilitates tumour vascularisation [52] and induces epithelial-to-mesenchymal transition through activation of PI3K–AKT and MAPK/ERK pathways in various cancers [53]. Serum IL-8 levels have also been shown to reflect tumour burden and therapy response in multiple malignancies such as NSCLC, prostate cancer, melanoma and renal cell carcinoma [54]. In addition, studies have revealed that cancer-associated fibroblasts secrete IL-6 into the NSCLC TME, resulting in chemoresistance [55] and increased metastatic potential [56]. IL-8 has also been shown to be released by infiltrating macrophages, contributing to increased tumour angiogenesis [57]. These results underscore the pro-tumorigenic signalling functions of IL-6 and IL-8 in the TME. IL-10 has pleiotropic functions which include regulation of cell proliferation, apoptosis and angiogenesis [58]. In NSCLC, IL-10 is considered to be pro-tumoral and immunosuppressive [59]. These biological roles could serve as rationale for the biomarker capacity shown by the cytokines at tumour progression in our cohort. We also noted that the presence of *TP53* mutations was associated with higher levels of serum IL-6 and IL-8, while the presence of *EML4-ALK* V3 was associated with significantly elevated serum IL-6 and IL-10. These observations indicate that these cytokines are biologically linked to higher tumour aggressiveness, as *EML4-ALK* V3 and *TP53* mutations detected either at baseline or at progression are established molecular risk factors of ALK + NSCLC according to several retrospective studies and the prospective ALTA-1L Phase 3 trial [60–63]. Future studies are required to functionally characterise the cytokines in this context.

The biomarker utility of cytokines is inherently limited as these molecules also mediate inflammation brought about by infection or tissue damage, and their systemic levels could be affected by corticosteroid therapy [64]. Indeed, there were moderate correlations between CRP, IL-6 and IL-10 in our dataset which possibly confounded data interpretation. This was not the case for IL-8, which is associated poorly with systemic and acute inflammation markers. Furthermore, IL-8 was the most significant progressive disease classifier among the three cytokines based on GLMM analysis, and thus the most useful. Nonetheless, careful assessment of the clinical profile of the patient must be considered in future studies.

While IL-6, IL-8 and IL-10 were significantly increased at disease progression, their baseline serum levels were not prognostic of the durable benefit of TKI therapy. This is in contrast to what has been reported in melanoma and NSCLC under immune checkpoint inhibitor therapy [43, 54]. This highlights the function of the cytokines related to innate immune activation in the TME, which is less important in predicting the success of tyrosine kinase inhibition.

Our analysis revealed that while the three interleukins paralleled the relative abundance of ctDNA longitudinally—and thus could be useful for tumour monitoring—correlations between them and ctDNA parameters were weak, suggesting independent contributions to tumour-specific information. Ideally, the combination of independent analytes should result in an enhanced capacity as biomarkers [15]. We tested this principle by modelling combinations of ΔIL-8, Δt-MAD and ΔVAF_mean_ in a binomial GLMM analysis and determining which set of classifiers resulted in the maximum AUC value for discriminating stable and progressive disease. Δt-MAD and ΔVAF_mean_ had comparable AUCs to our previously reported values on the same cohort [16]. Notably, the addition of ΔIL-8 to ΔVAF_mean_ (i.e., mutational data) resulted in significantly increased AUC. SNV detection is highly dependent on the tumour fraction in plasma DNA, and it is well-known that inter-individual ctDNA shedding is variable [15, 43]. Our results suggest that cytokine analysis could augment this limitation since detection is possible for all samples. The addition of ΔIL-8 did not significantly improve the AUC_Δt-MAD_. Even so, both parameters were non-redundant classifiers that significantly impacted the model outcome. The AUC generated using the combination of ΔIL-8, Δt-MAD and ΔVAF_mean_ resulted in the optimum value. However, this model did not significantly outperform the bivariate model derived from the ctDNA parameters, indicating the superiority of ctDNA as markers of tumour progression. Nonetheless, our study provides basis for further studies on the utility of cytokines as liquid biopsy analytes for disease monitoring in ALK + NSCLC. Serum cytokines are easily accessible and their detection requires less economic and technical resources, making them attractive candidates as biomarkers.

Our study is limited by the small and heterogeneous nature of the patient cohort in terms of TKI therapies and their order of administration per patient. Future studies should also consider the clinical history of patients in terms of health conditions impacting systemic inflammation. Additional serum cytokines could also be screened for potential disease monitoring utility, including TGF-β, which has been demonstrated in a preclinical study to have abrogated tumour suppressive response in ALK + tumours [65], and other serum cytokines reported [22, 30] to be associated with NSCLC. Validation of our results in a larger and prospective cohort is necessary to determine whether the addition of cytokine evaluation could be an improvement to current tumour monitoring modalities in the clinical setting.

## Supplementary information

Supplemental figure 1

Supplemental figure 2

Supplemental figure 3

Supplemental figure 4

Supplemental figure 5

Supplemental figure 6

Supplemental figure 7

Supplemental figure 8

## Data Availability

The data generated in this study are available from the corresponding author upon reasonable request.
