# Supplementary material for: Integrated circulating tumour DNA and cytokine analysis for therapy monitoring of ALK-rearranged lung adenocarcinoma
Source: Br J Cancer. 2023 Apr 29;129(1):112–21. doi: 10.1038/s41416-023-02284-0 (PMC10307797; doi:10.1038/s41416-023-02284-0)
Supplement: Supplementary file 1 — Supplemental figure 1 [file 41416_2023_2284_MOESM1_ESM.pdf]

Supplemental figure 1

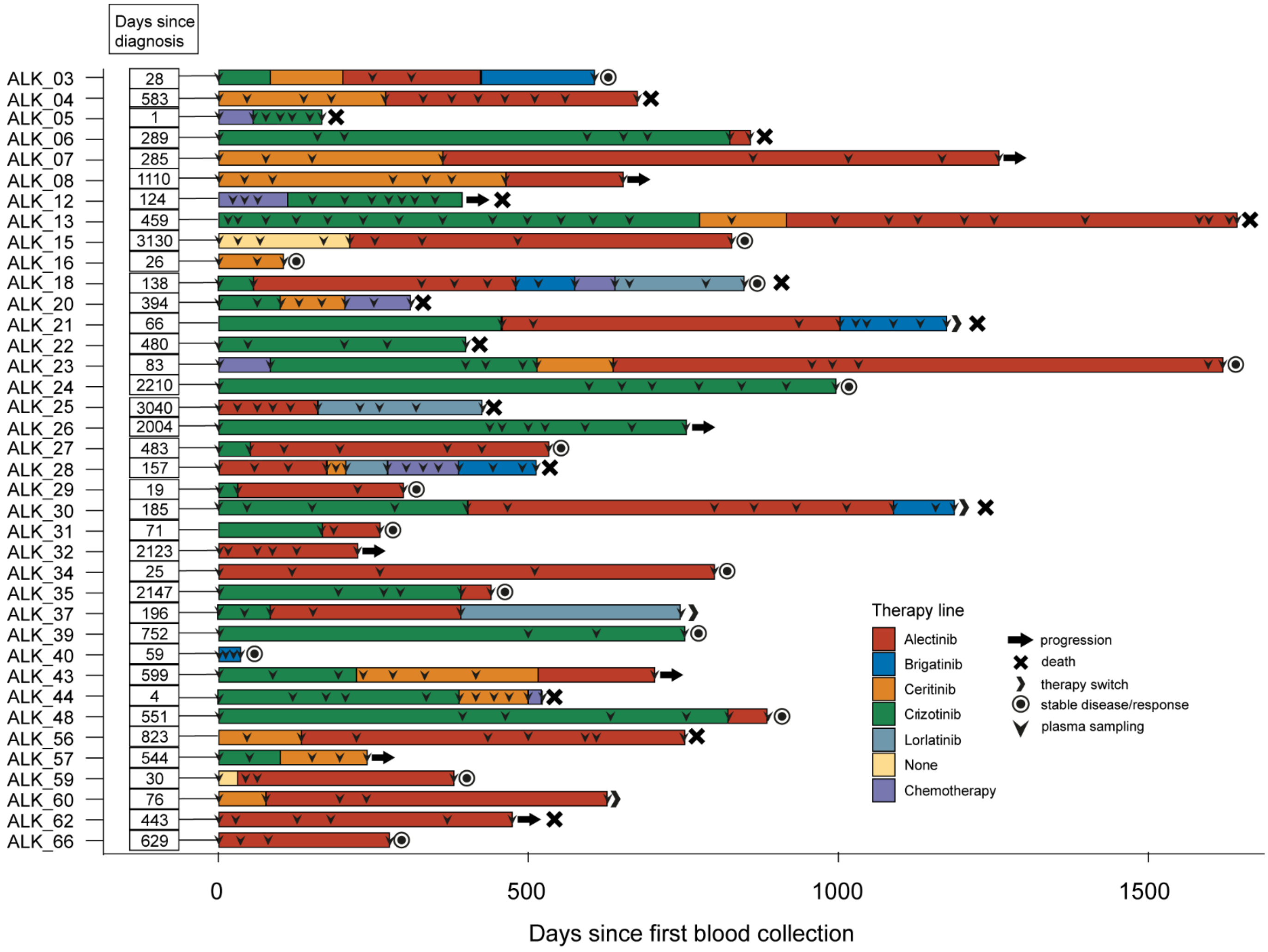

Supplemental figure 1. Timeline of longitudinal serum collection in patients of the ALK+ NSCLC cohort.
