# Supplementary material for: Integrated circulating tumour DNA and cytokine analysis for therapy monitoring of ALK-rearranged lung adenocarcinoma
Source: Br J Cancer. 2023 Apr 29;129(1):112–21. doi: 10.1038/s41416-023-02284-0 (PMC10307797; doi:10.1038/s41416-023-02284-0)
Supplement: Supplementary file 2 — Supplemental figure 2 [file 41416_2023_2284_MOESM2_ESM.pdf]

Supplemental figure 2

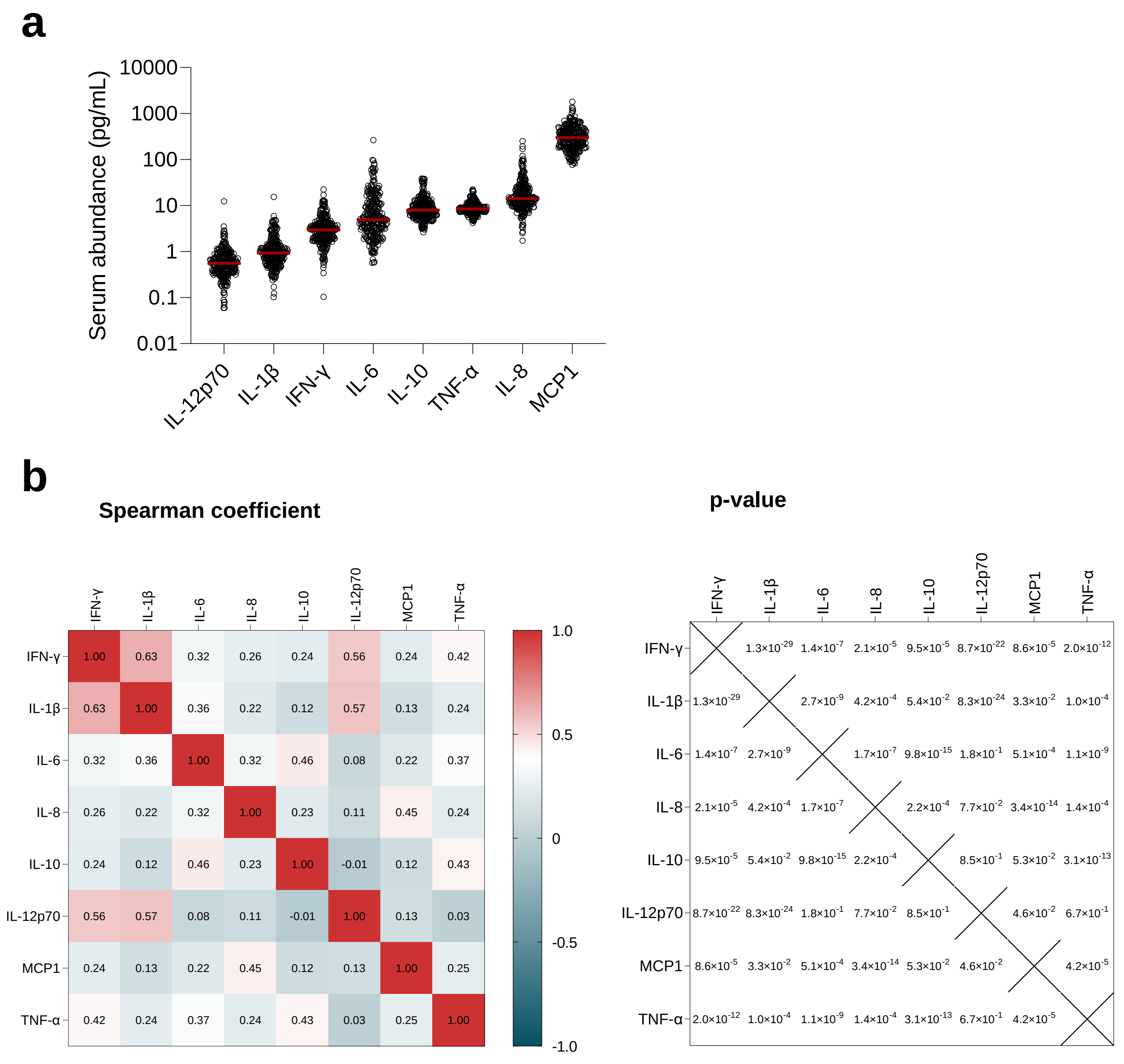

Supplemental figure 2. **a** The range of measured levels of cytokines in all samples included in the study. **b** Spearman correlation coefficient matrix (left) and corresponding p-values (right) showing associations between tested cytokines.
