# Supplementary material for: Integrated circulating tumour DNA and cytokine analysis for therapy monitoring of ALK-rearranged lung adenocarcinoma
Source: Br J Cancer. 2023 Apr 29;129(1):112–21. doi: 10.1038/s41416-023-02284-0 (PMC10307797; doi:10.1038/s41416-023-02284-0)
Supplement: Supplementary file 3 — Supplemental figure 3 [file 41416_2023_2284_MOESM3_ESM.pdf]

Supplemental figure 3

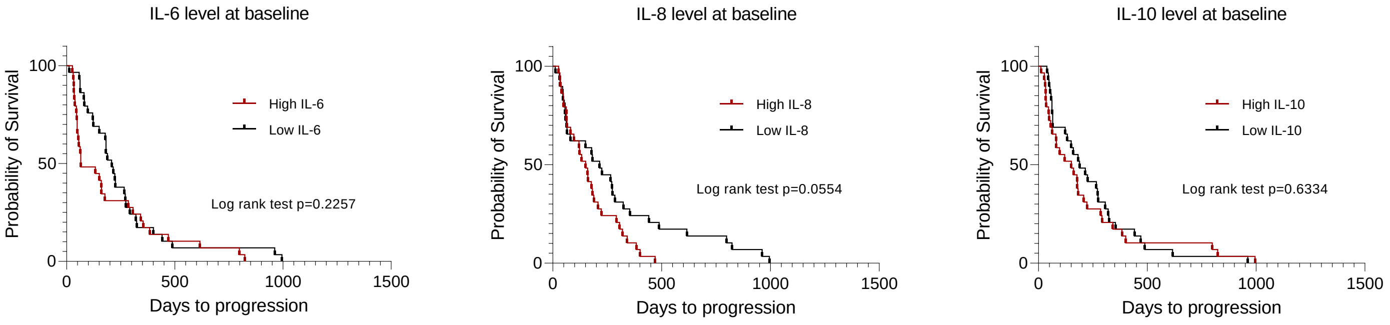

Supplemental figure 3. Progression-free survival plots showing that baseline serum cytokine levels were not indicative of durability of therapy. The median cytokine value was taken as the threshold between high and low groups.
