# Supplementary material for: Integrated circulating tumour DNA and cytokine analysis for therapy monitoring of ALK-rearranged lung adenocarcinoma
Source: Br J Cancer. 2023 Apr 29;129(1):112–21. doi: 10.1038/s41416-023-02284-0 (PMC10307797; doi:10.1038/s41416-023-02284-0)
Supplement: Supplementary file 4 — Supplemental figure 4 [file 41416_2023_2284_MOESM4_ESM.pdf]

Supplemental figure 4

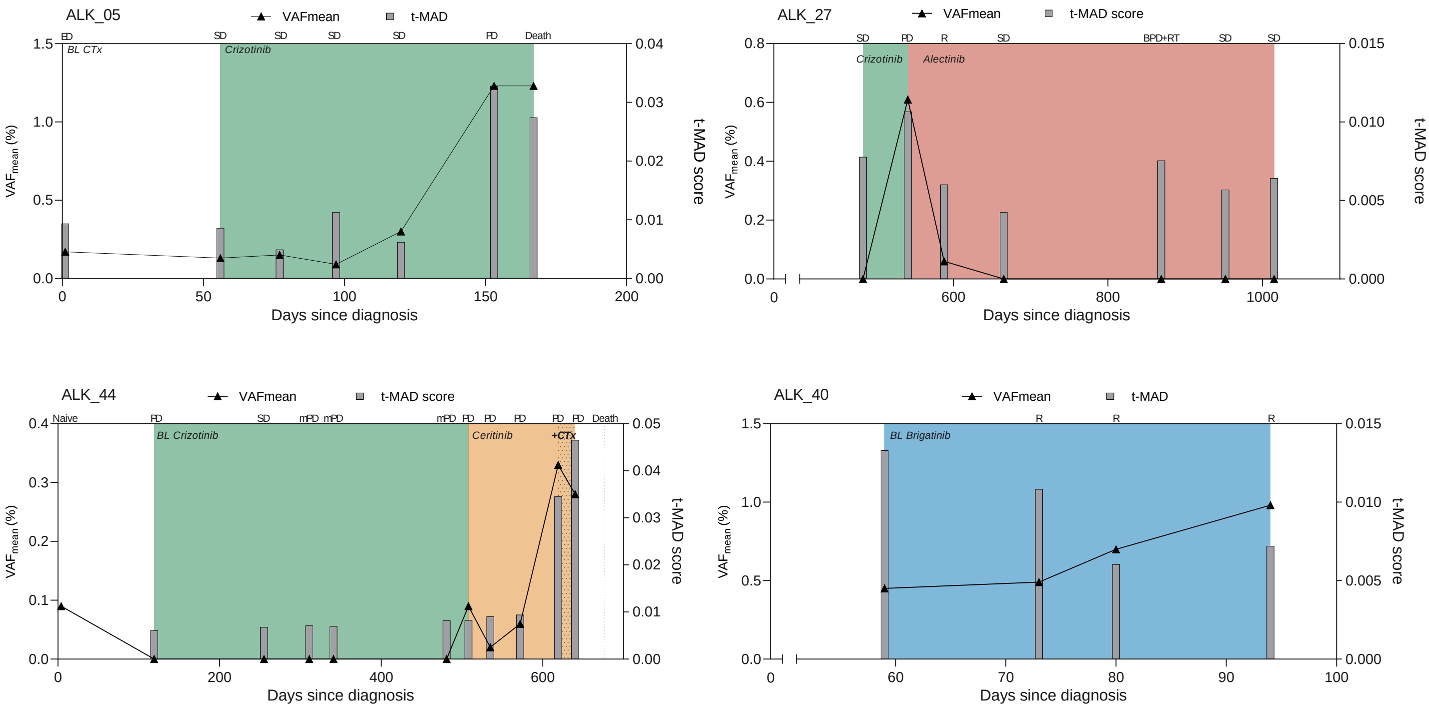

Supplemental figure 4. Longitudinal ctDNA kinetics of representative ALK+ NSCLC patients. CTx: chemotherapy; SD: stable disease; PDTC: progressive disease with therapy change; mPD: metastatic progressive disease; BPD: brain progressive disease; RT: radiotherapy; R: response.
