# Supplementary material for: Integrated circulating tumour DNA and cytokine analysis for therapy monitoring of ALK-rearranged lung adenocarcinoma
Source: Br J Cancer. 2023 Apr 29;129(1):112–21. doi: 10.1038/s41416-023-02284-0 (PMC10307797; doi:10.1038/s41416-023-02284-0)
Supplement: Supplementary file 5 — Supplemental figure 5 [file 41416_2023_2284_MOESM5_ESM.pdf]

Supplemental figure 5

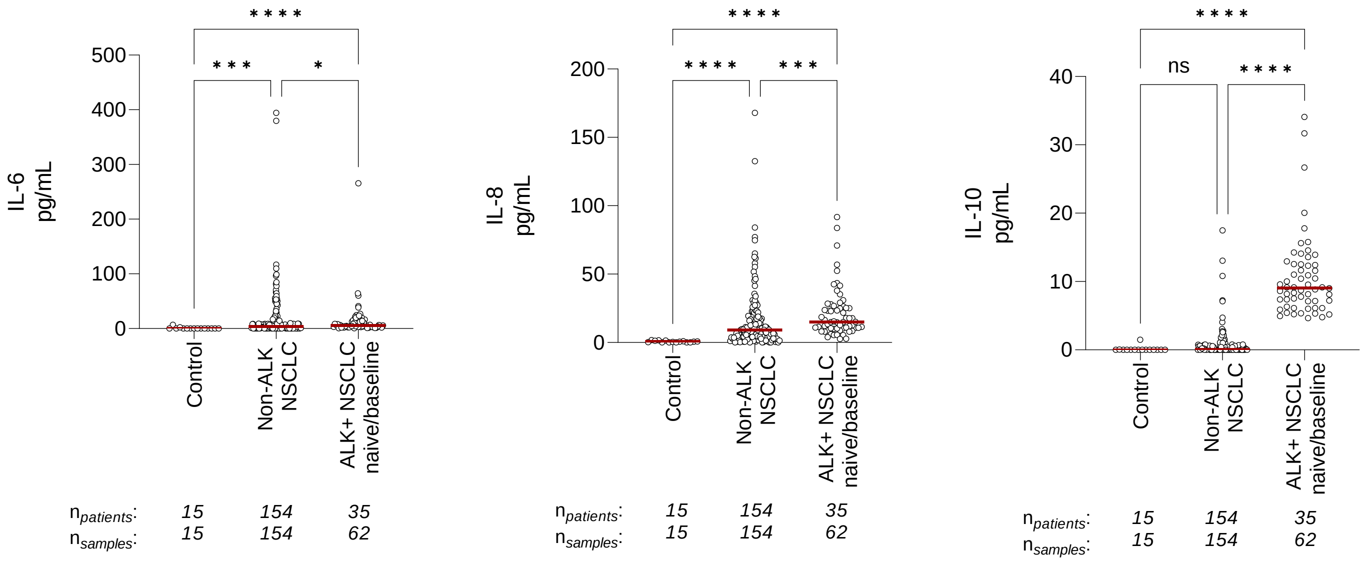

Supplemental figure 5. Comparisons of serum cytokine levels of healthy controls, non-ALK NSCLC patients at therapy baseline, and ALK+ NSCLC patients that are treatment naïve or at therapy baseline. Data for healthy controls and non-ALK NSCLC patients were retrieved from reference #22. The median of each group is shown by the red line. Patient counts and corresponding serum samples used in each group are indicated below the x-axis. Statistical significance was tested using nonparametric Kruskal-Wallis test followed by multiple comparisons, ns: not significant; **P*<0.05; ***P*<0.01; ****P*<0.001; *****P*<0.0001.
