# Supplementary material for: Integrated circulating tumour DNA and cytokine analysis for therapy monitoring of ALK-rearranged lung adenocarcinoma
Source: Br J Cancer. 2023 Apr 29;129(1):112–21. doi: 10.1038/s41416-023-02284-0 (PMC10307797; doi:10.1038/s41416-023-02284-0)
Supplement: Supplementary file 6 — Supplemental figure 6 [file 41416_2023_2284_MOESM6_ESM.pdf]

Supplemental figure 6

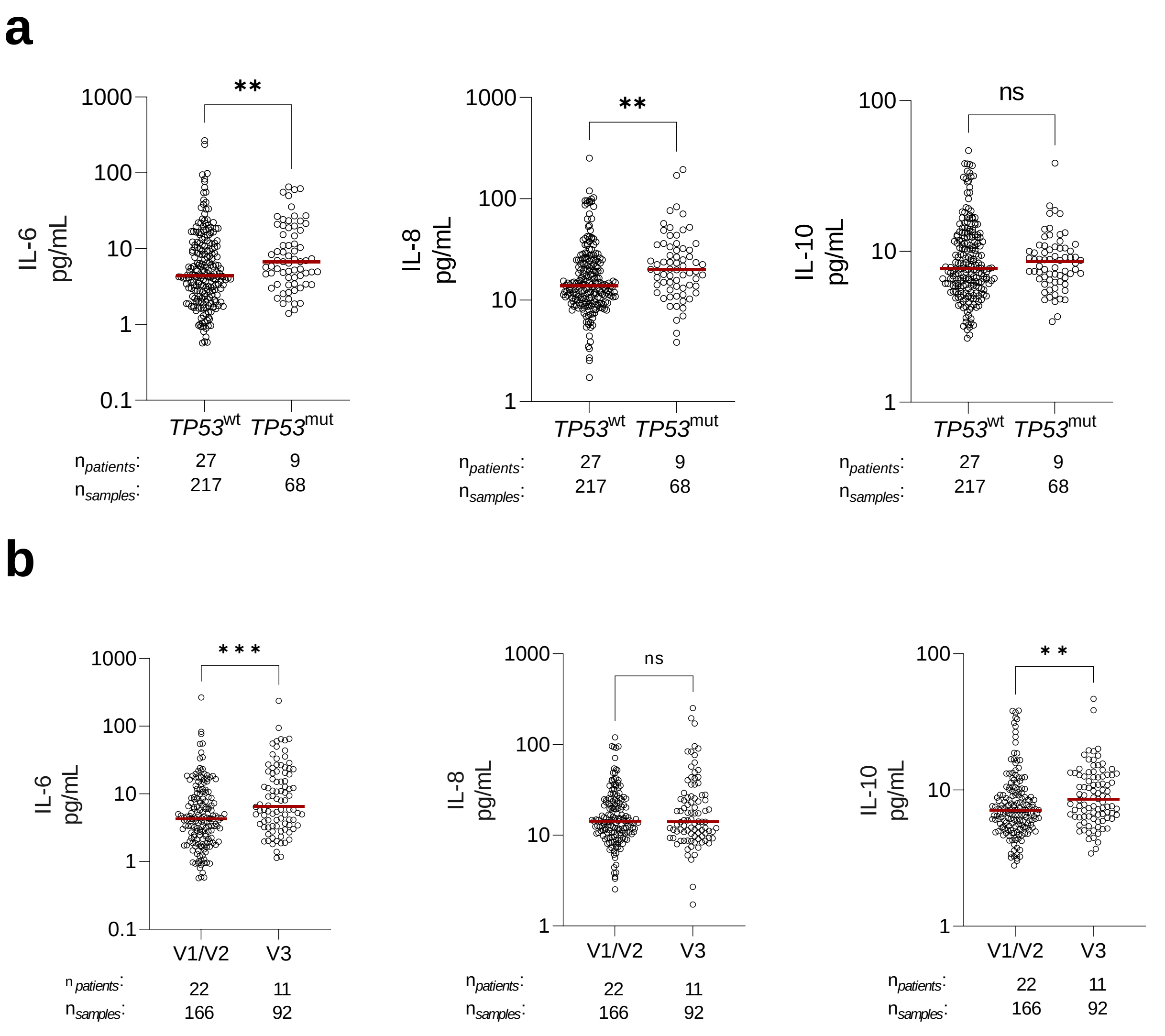

Supplemental figure 6. **a** Higher levels of IL-6 and IL-8 were measured in serum samples originating from patients with *TP53*-mutant tumors. **b** Higher levels of IL-6 and IL-10 were measured in serum samples originating from patients harboring *EML4-ALK* fusion variant 3 (V3). Patient counts and corresponding serum samples used in each group are indicated below the x-axis. Statistical significance was evaluated using Mann Whitney U test. Each dot represents the median of duplicate measurements per sample. The median of each group is shown by the red line. ns: not significant; **P*<0.05; ***P*<0.01; ****P*<0.001.
