# Supplementary material for: Integrated circulating tumour DNA and cytokine analysis for therapy monitoring of ALK-rearranged lung adenocarcinoma
Source: Br J Cancer. 2023 Apr 29;129(1):112–21. doi: 10.1038/s41416-023-02284-0 (PMC10307797; doi:10.1038/s41416-023-02284-0)
Supplement: Supplementary file 7 — Supplemental figure 7 [file 41416_2023_2284_MOESM7_ESM.pdf]

Supplemental figure 7

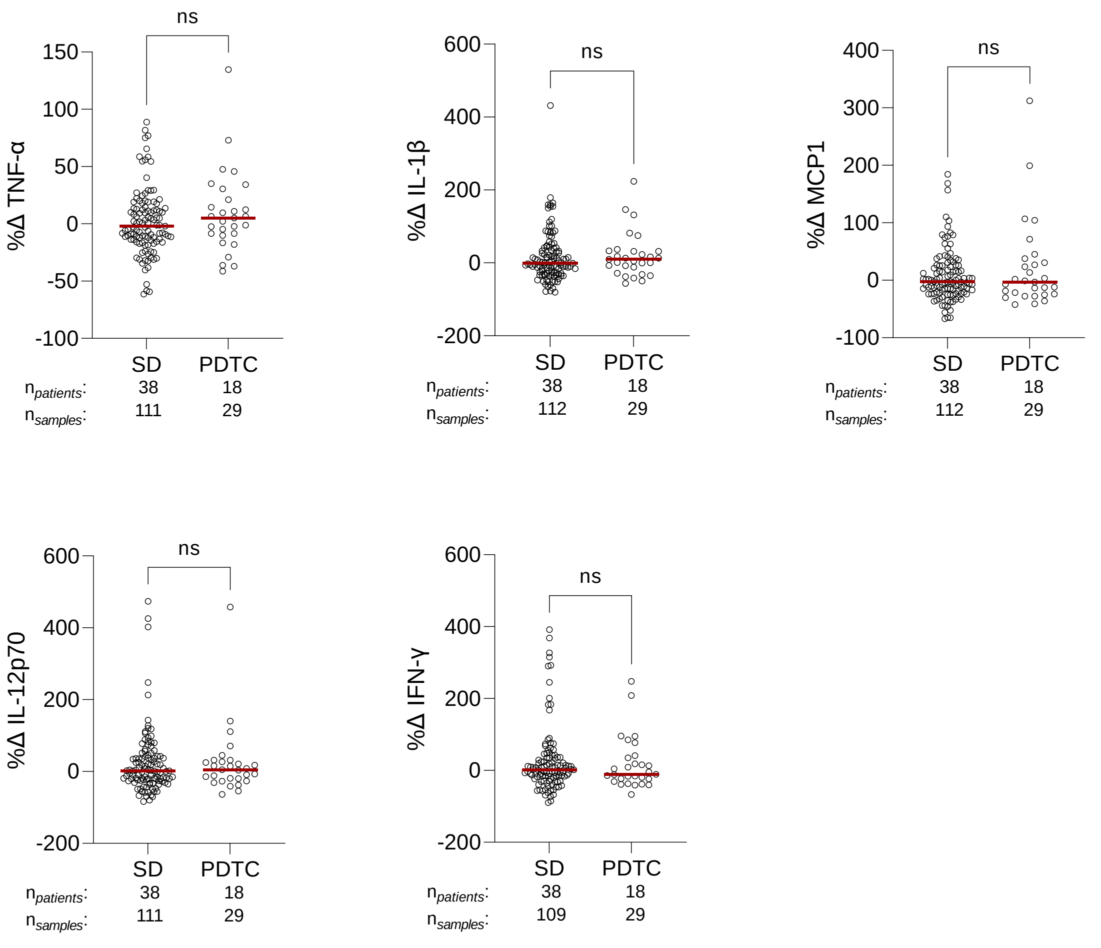

Supplemental figure 7. Changes in the serum cytokine levels of TNF-α, IL-1β, MCP1, IL-12p70 and IFN-γ were not significantly different between stable and progressive disease. The median of each group is shown by the red line. Patient counts and corresponding serum samples used in each group are indicated below the x-axis. Statistical significance was tested using Mann Whitney U test, ns: not significant.
