# Supplementary material for: Integrated circulating tumour DNA and cytokine analysis for therapy monitoring of ALK-rearranged lung adenocarcinoma
Source: Br J Cancer. 2023 Apr 29;129(1):112–21. doi: 10.1038/s41416-023-02284-0 (PMC10307797; doi:10.1038/s41416-023-02284-0)
Supplement: Supplementary file 8 — Supplemental figure 8 [file 41416_2023_2284_MOESM8_ESM.pdf]

Supplemental figure 8

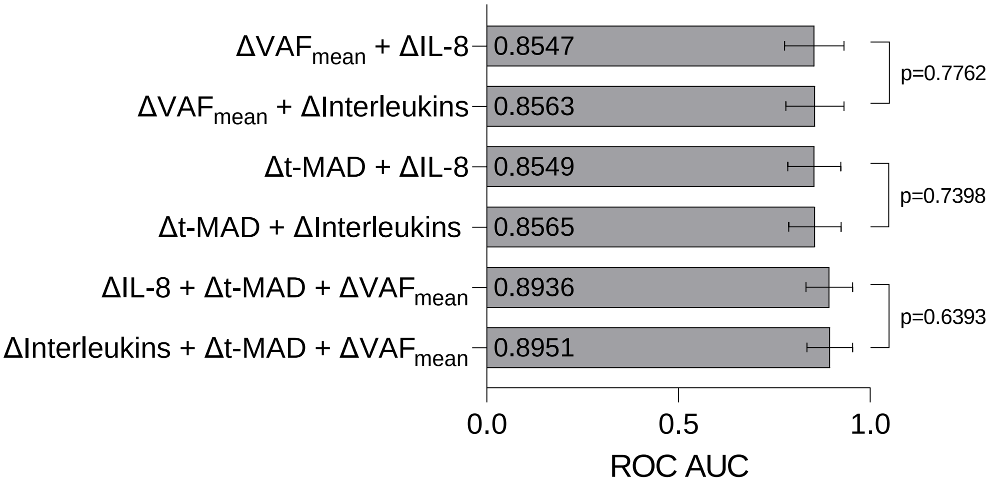

Supplemental figure 8. Area under the curve (AUC) values of receiver operating characteristic (ROC) curves distinguising stable and progressive disease in ALK+ NSCLC using independent and combinatorial liquid biopsy parameters. Pairwise AUC significance test was performed using the DeLong method. ΔInterleukins: ΔIL-6 + ΔIL-8 + ΔIL-10. Error bars indicate the 95% confidence intervals.
